# Supplementary material for: Identifying risk factors for cancer-specific early death in patients with advanced endometrial cancer: A preliminary predictive model based on SEER data
Source: PLoS One. 2025 Feb 12;20(2):e0318632. doi: 10.1371/journal.pone.0318632 (PMC11819511; doi:10.1371/journal.pone.0318632)
Supplement: S1 Table — (DOCX) [file pone.0318632.s001.docx]

**S1 Table.**The variance inflation factor values of each variable

GVIF Df GVIF^(1/(2*Df))

Age 1.165 2 1.039

Race 1.126 3 1.020

Marital_status 1.051 1 1.025

Tumor_Size 1.158 2 1.037

classification 1.665 2 1.136

grade 2.240 3 1.144

T 1.695 2 1.141

N 1.534 3 1.074

M 1.686 1 1.298

Surgery 2.717 3 1.181

Lymph_node 1.139 1 1.067

Radiation 1.209 1 1.099

Chemotherapy 5.418 1 2.328

Systemic 6.756 1 2.599

Bone 1.142 1 1.069

Brain 1.043 1 1.021

Liver 1.125 1 1.061

Lung 1.392 1 1.180
