# Supplementary material for: Identifying risk factors for cancer-specific early death in patients with advanced endometrial cancer: A preliminary predictive model based on SEER data
Source: PLoS One. 2025 Feb 12;20(2):e0318632. doi: 10.1371/journal.pone.0318632 (PMC11819511; doi:10.1371/journal.pone.0318632)
Supplement: Attachment — Submitted filename: Response to Reviewers.docx [file pone.0318632.s003.docx]

**Response to Reviewers**

**Reviewer #1: The manuscript reports on an interesting study that collected clinical data from patients with advanced endometrial cancer in the SEER database from 2018 to 2021, randomly dividing all patients into a training cohort and a validation cohort in a ratio of 7:3. Multivariate logistic regression analysis was performed on the training cohort to screen out the risk factors of cancer-specific early death in patients with advanced endometrial cancer, and the nematogram model was further constructed for verification. However, there are still some problems in this study, the flow chart is rough and some arrows point wrong. In addition to the marital status of single and married patients, are there any other conditions, such as divorce and widowhood, etc., in the general information of patients? Are these people included in the study?**

Thank you for your thoughtful comments regarding our manuscript. We appreciate your insights and have made the following revisions to address your concerns:

Flow Chart Revision: We have revised the flow chart to ensure that all arrows accurately represent the patient selection process and the study flow. The updated chart clearly indicates the sequence of steps from data extraction to final analysis, along with the number of patients at each stage.

Marital Status Clarification: In our study, "Married" refers to individuals in legal marriages or recognized common-law partnerships. The "Single" category encompasses all individuals who are unmarried, including those who are divorced or widowed. We have added this clarification to the manuscript for greater transparency.

We also included a note in the "Data Collection" section defining marital status:

Marital Status: "Married" for individuals in legal or common-law marriages, and "Single" for individuals who are unmarried, divorced, or widowed.

These revisions aim to enhance the clarity of our patient cohorts and address the methodological concerns you raised. We believe these changes will improve the overall quality of our manuscript.

Thank you again for your thorough review. If you have any further questions or require additional information, please feel free to reach out.

**Reviewer #2: Thank you for introducing such a good and reliable method for predicting the mortalities associated with endometrial cancer which will be positively reflected on the councelling as well as managing those patients.**

Thank you for your positive and encouraging comments regarding our nomogram for predicting mortalities associated with endometrial cancer. We are thrilled to hear that you found our method reliable and impactful for patient counseling and management.

Your feedback serves as a great motivation for us and reinforces our commitment to addressing the needs of the medical community. We are optimistic that our nomogram will be a valuable tool in clinical decision-making, helping healthcare providers and patients make more informed choices regarding treatment and prognosis.

We remain dedicated to advancing research in this field and will continue to seek ways to refine and enhance our predictive models. Thank you once again for your kind words and recognition of our work’s potential to improve patient care.

**Reviewer #3: 1. This is a clear and concise article based on correct statistical methods on predicting cancer-specific early death in patients with advanced endometrial cancer.**

Thank you for your kind words and positive evaluation of our article. We appreciate your recognition of the importance of clear and concise analytical methods in predicting cancer-specific early deaths in patients with advanced endometrial cancer. Your feedback validates the accuracy and reliability of our research, encouraging us to continue our efforts to meet the needs of the medical community and improve patient care.

**2. While details explanation on main statistical methods were presented, there was no mention regarding any other clinical prediction model in the literature for advanced endometrial cancer. If there are other models, this should be mentioned in the introduction and compared in the discussion section. If none, this should also be mentioned that there is no prediction model for this specific patient’s group. There should be some information regarding prediction model in the introduction section.**

We would like to express our sincere gratitude for your meticulous review and valuable feedback. Your observation regarding the absence of mention of other clinical prediction models in the literature for advanced endometrial cancer is well-taken and highlights an oversight in our manuscript. We acknowledge the importance of referencing existing prediction models in the literature and comparing them in the discussion section to provide readers with a comprehensive understanding of the context and significance of our study.

Upon re-examining the relevant literature, we regret to find that, to the best of our knowledge, there are currently no other established clinical prediction models specifically for this patient group with advanced endometrial cancer. This may be attributed to the complexity and heterogeneity of the disease, which poses challenges in developing a universally applicable prediction model. However, this realization also presents our study with a unique opportunity to develop the first prediction model tailored to this specific patient cohort, thereby addressing a gap in the existing literature.

In response to your concern, we have added the following statement to the introduction section of our paper: " It should be highlighted that the current literature does not offer comprehensive predictive models specifically designed for patients with advanced endometrial cancer."

Additionally, in the discussion section, we have included a reflection on this state of affairs: "Given the current dearth of prediction models for patients with advanced endometrial cancer, our study may serve as a pioneering effort. We encourage future researchers to continue exploring and validating our model and to build upon it for further development."

We believe these additions will enhance the completeness and transparency of our article. We appreciate your valuable comments and look forward to your further feedback.

**3. The methods section does not specify the coding of the outcome variable for logistic regression: Was it early death or cancer-specific early death?**

Thank you for your careful review and insightful comments on our manuscript. You are correct in noting that we did not explicitly define whether "early death" or "cancer-specific early death" was used as the outcome variable in our logistic regression analysis within the methods section. This is indeed a critical detail that requires clarification.

In our study, we utilized "cancer-specific early death" as the outcome variable for our logistic regression model. This was defined as death due to endometrial cancer within six months of the initial pathological diagnosis. To address your concern and to avoid any ambiguity, we will revise the methods section to include a clear definition of our outcome variable.

Here is how we propose to revise the methods section to include this definition:

" The primary outcome variable for the logistic regression analysis was defined as 'cancer-specific early death,' which refers to death attributable to endometrial cancer within six months following the initial pathological diagnosis. "

By specifying our definition of 'cancer-specific early death' in this manner, we aim to enhance the clarity and transparency of our methods and to ensure that the outcome variable is clearly understood by readers. We believe this revision will address your concern and strengthen our manuscript.

We appreciate your valuable feedback and are grateful for the opportunity to improve the quality of our submission.

**4. Table 1 shows that there is no variation between training and validation data sets in terms of considered variable and hence model performance was good in validation. However, it does not validate this model in real life heterogenous data set, which is more common.**

We greatly appreciate your insightful comments and acknowledge the critical importance of external validation in the context of our predictive model for advanced endometrial cancer. Your feedback has prompted us to re-evaluate our approach and focus on the aspects that are most relevant to our study's objectives.

In light of the limitations you've pointed out, particularly the absence of detailed comorbidity data in the SEER database, we have decided to pivot our manuscript to emphasize the identification and analysis of key risk factors associated with early cancer-specific mortality in patients with advanced endometrial cancer, rather than the model's predictive performance.

Here are the adjustments we have made to address your concerns:

1. Refocused Manuscript Scope: We have revised the manuscript to underscore the importance of risk factors such as tumor size, histological type, tumor grade, and clinical stage. While these factors do not encompass the full spectrum of comorbidities, they remain significant for clinical decision-making and treatment strategy formulation.

2. Acknowledgment of Preliminary Nature: We have explicitly stated in the manuscript that our findings are preliminary and that our model serves as a foundation for future studies. This acknowledgment is prominently featured in the discussion section, setting the stage for more comprehensive analyses that will include comorbidity data.

3. Future Research Directions: We have outlined potential directions for future research, suggesting the incorporation of comorbidity data to enhance the model's predictive power and generalizability. We propose that future studies may benefit from multicenter, prospective designs or collaborative analyses with other databases to capture a more holistic view of patient health.

By refocusing on the risk factors and the preliminary nature of our findings, we aim to provide actionable insights for the clinical management of advanced endometrial cancer while also setting the stage for more robust validation studies in the future. We believe this approach aligns with the current data available and addresses the limitations inherent in our study design.

We are grateful for your valuable feedback and are committed to incorporating these considerations into our final manuscript. We are confident that these revisions will enhance the relevance and impact of our research within the scientific community.

**5. In line 210: The study found that the early all-cause mortality rate for stage III-IV endometrial cancer was 25.1%, while the early cancer-specific mortality rate was 6.8%. Therefore, a large percentage of early death of cancer patients are due to non-cancer related causes including co-morbidities, which were not included in the model.**

Thank you for highlighting this important aspect of our study. We appreciate your observation regarding the distinction between all-cause mortality and cancer-specific mortality in patients with stage III-IV endometrial cancer.

Specifically, The cancer-specific mortality rate for endometrial cancer is 6.8%，while the 25.1% all-cause mortality includes deaths related to other cancers and co-morbid factors.You are correct that a significant proportion of early deaths may be attributed to non-cancer related causes, including co-morbidities, which are not accounted for in our current model. This is indeed a limitation of our study, primarily due to the lack of detailed comorbidity data in the SEER database.

To address this limitation, we have taken the following steps:

Emphasis on Risk Factors: We have adjusted the manuscript to emphasize the identification of risk factors associated with cancer-specific early death. While our model does not include co-morbidities, we have focused on the risk factors that are available and can be used to inform clinical decision-making.

Acknowledgment of Limitations: We have explicitly acknowledged this limitation in the discussion section of our manuscript. We have clarified that the model's predictive ability may be limited in real-world settings where co-morbidities play a significant role in patient outcomes.

Future Research Directions: We have proposed that future research should aim to incorporate comorbidity data to enhance the model's predictive accuracy and applicability. This could involve multicenter, prospective studies or collaborations with other databases that contain more comprehensive patient health information.

Model Refinement: We are committed to refining our model in future studies by including additional variables and adjusting parameters to better capture the impact of co-morbidities on early mortality rates.

We appreciate your feedback and will ensure that these points are clearly articulated in the final version of our manuscript. We believe that by addressing this limitation and focusing on the risk factors we can control and measure, our research can still provide valuable insights into the early mortality rates of patients with advanced endometrial cancer.

**6. Co-morbidity data were absent in the used data set, which is a big limitation of this article in terms of the usefulness of this predictive model. Previous studies showed that comorbidity indices like Age-adjusted Charlson Comorbidity Index is a strong predictor of overall survival of early-stage endometrial cancer patients. (Al Feghali KA, Robbins JR, Mahan M, Burmeister C, Khan NT, Rasool N, Munkarah A, Elshaikh MA. Predictive Capacity of 3 Comorbidity Indices in Estimating Survival Endpoints in Women With Early-Stage Endometrial Carcinoma. Int J Gynecol Cancer. 2016 Oct;26(8):1455-60. doi: 10.1097/IGC.0000000000000802. PMID: 27488218). This article identifies the significant risk factors for early-stage endometrial cancer but has limited use as a prediction model. Therefore, the focus of this article could be more on identifying risk factors over prediction unless comorbidity variables are included in the models.**

We sincerely appreciate the keen observation you've made regarding the absence of comorbidity data in our study, a point that resonates with the heart of our research's limitations. Your reference to the pivotal role of comorbidity indices like the Age-adjusted Charlson Comorbidity Index in predicting survival outcomes is both apt and enlightening.

In response to your critique, we've taken a novel approach to reframe our manuscript. Rather than presenting our findings as a predictive model, we've chosen to pivot towards a narrative that emphasizes the discovery and elucidation of risk factors associated with early mortality in advanced endometrial cancer. This shift allows us to sidestep the limitations imposed by the lack of comorbidity data in the SEER database, focusing instead on what our data can robustly support.

Here are the innovative steps we've taken to address your concerns:

1. Narrative Shift: We've reframed our manuscript to tell a story of discovery, focusing on the identification of risk factors rather than the prediction of outcomes. This narrative shift allows us to highlight the importance of our findings without overstepping the bounds of our data.

2. Risk Factor Exploration: We've delved deeper into the identified risk factors, exploring their mechanistic roles and potential interactions. This approach not only adds depth to our analysis but also provides a foundation for future studies to build upon.

3. Limitation Embracement: Instead of viewing the absence of comorbidity data as a mere limitation, we've embraced it as an opportunity to call for a more holistic approach to data collection in future studies. We believe this proactive stance can inspire a new wave of research that addresses these gaps.

4. Vision for the Future: We've crafted a vision for future research that goes beyond the inclusion of comorbidity data. We propose a multi-faceted approach that includes not only the integration of comorbidity indices but also the exploration of genetic, lifestyle, and environmental factors that may influence survival outcomes.

5. Model as a Springboard: We position our model not as the final word but as a springboard for future research. Our hope is that it will inspire others to take up the challenge of refining and expanding upon our work, ultimately leading to more accurate and comprehensive predictive models.

By taking these innovative steps, we aim to transform the limitations of our study into a catalyst for future research. We believe that this approach not only addresses your concerns but also adds a unique and compelling dimension to our manuscript.

Thank you once again for your valuable feedback. We are excited about the potential of our revised manuscript to contribute to the field and look forward to any further insights you may have.

**7. P in P-value should be italic.**

Thank you for pointing out the importance of typographical accuracy in scientific writing. You are correct that the "P" in "P-value" should be italicized to distinguish it as a statistical term. This convention is important for maintaining clarity and consistency in scientific communication.

We appreciate your attention to detail and will ensure that the "P" in "P-value" is italicized throughout the manuscript to adhere to the standard typographical guidelines for statistical symbols and terms.

**8. CI in Table 2 should have either – sign or comma but not Tilda sign (~).**

Thank you for your careful review and for pointing out the inconsistency in the use of symbols in Table 2. You are correct that the confidence interval (CI) should be denoted with an en dash (–) or a comma, rather than a tilde (~), to maintain standard scientific notation and ensure clarity.

We apologize for the oversight and appreciate your attention to this detail. We will correct the symbols in Table 2 to use an en dash for the CI, which is the appropriate symbol for indicating a range, as per standard typographical conventions in scientific writing .

Thank you again for your valuable feedback. We will make the necessary revisions to ensure that our manuscript adheres to the highest standards of clarity and precision.

**9. In line 118: “An ROC curve closer to 1 reflects a model with high TPR and low FPR, making it a better-performing model”. Sentences like this may be deleted depending on the background of the readers of this article.**

Thank you for your feedback on the manuscript. You've pointed out that the sentence on line 118, which describes the interpretation of the ROC curve, may not be necessary depending on the background of the readership. This is a valid consideration, as the audience for our article may already be familiar with the basics of ROC analysis.

To address your comment, we have decided to revise the manuscript. We will remove the sentences like on line 118 to avoid redundancy and assume a level of prior knowledge among our readers that is appropriate for the journal's scope. We believe this change will tighten the narrative flow and focus the article more directly on our novel findings and contributions.

We appreciate your insight and are confident that this revision will make the article more concise and suitable for the intended audience.

Thank you again for your helpful feedback.
