# Supplementary material for: Identifying risk factors for cancer-specific early death in patients with advanced endometrial cancer: A preliminary predictive model based on SEER data
Source: PLoS One. 2025 Feb 12;20(2):e0318632. doi: 10.1371/journal.pone.0318632 (PMC11819511; doi:10.1371/journal.pone.0318632)
Supplement: Attachment — Submitted filename: Response_to_Reviewers_auresp_2.docx [file pone.0318632.s004.docx]

**Response to Reviewers**

**Reviewer #3: Thank you for applying my comments correctly. I have two further comments:**

**1. In the Results section of the Abstract, active voice was used whereas passive voice was used throughout the article. It is better to be consistent.**

Dear Reviewer,

Thank you for your careful review and valuable feedback on our manuscript. We greatly appreciate your observation regarding the inconsistency in voice usage between the Results section of the Abstract and the rest of the article. We agree that maintaining consistency is crucial for the clarity and coherence of the manuscript.

In response to your comment, we have revised the Abstract section to ensure that the passive voice is used consistently throughout the article. This adjustment aligns with the overall style of the manuscript and enhances its uniformity.

Once again, we sincerely thank you for your constructive suggestion, which has helped us improve the quality of our work.

**2. First paragraph of the discussion section seems better fit in the methods section of the article; rather than a matter of discussion of results/findings.**

Dear Reviewer,

Thank you for your careful review and constructive feedback on our manuscript. We greatly appreciate your observation that the first paragraph of the Discussion section would be more appropriately placed in the Methods section. We agree that this adjustment improves the logical flow and structure of the manuscript.

In response to your comment, we have relocated the definition of early mortality to the Methods section. Additionally, we have removed this content from the Discussion section to ensure clarity and focus on the interpretation of results.

Once again, we sincerely thank you for your valuable suggestion, which has helped us enhance the organization and quality of our work.

**Reviewer #4: Based on the seer database, the characteristics of diseases were analyzed in large samples to provide clinical diagnosis and treatment strategies and guide clinical treatment. However, the innovation of this paper is insufficient. There are a large number of published studies on the characteristics of endometrial cancer in the seer database. However, the conclusion does not have much significance for the clinical work.**

Thank you for your thoughtful feedback regarding the innovation and clinical significance of our study.

Regarding the innovation of our study:

We acknowledge that there are numerous published studies analyzing the characteristics of endometrial cancer based on the SEER database. However, our study focuses specifically on advanced endometrial cancer, particularly on early mortality in this population, which has been less explored in the literature. By identifying clinically accessible and relevant risk factors, our findings provide valuable insights that can aid in informed decision-making for patient management.

Regarding the clinical significance:

Our study aims to address a critical gap in the management of advanced endometrial cancer. The identified risk factors assist clinicians in the early identification of high-risk patients, enabling timely interventions and tailored treatment strategies. Furthermore, we have developed a predictive model for early mortality in advanced endometrial cancer. Given the current dearth of such prediction models, our study serves as a pioneering effort in this area. We hope that our model will provide a scientific foundation for future research and encourage other researchers to validate and build upon it for further development.

We believe that these aspects highlight the novelty and clinical relevance of our work. Once again, we sincerely thank you for your valuable suggestions, which have helped us refine and strengthen our manuscript.

**Reviewer #5: 1. The study presents a cohort study to identify key risk factors for cancer-specific early death in advanced endometrial cancer and develop a predictive nomogram. The treatment factor depends on clinical decisions of intervention. The addition of clinical guidelines or decision pathways can support applications.**

Thank you for your insightful comment regarding the inclusion of clinical guidelines or decision pathways to support the application of our findings.

1. Study Focus and Contribution: Our study focuses on identifying key risk factors for cancer-specific early death in advanced endometrial cancer and developing a predictive nomogram. We aimed to create a model based on accessible and clinically relevant variables to aid in early risk stratification and individualized treatment planning.

2. Clinical Decision Factors: We acknowledge that treatment decisions often depend on clinical judgment, patient preferences, and institutional practices. While the current study does not incorporate detailed clinical guidelines or decision pathways, we agree that these elements would enhance the applicability of the findings.

3. Future Research Directions: We propose that future studies should integrate clinical guidelines or decision pathways to validate and extend our nomogram’s utility. Collaborative efforts involving clinical experts and guideline developers can bridge the gap between statistical models and practical implementation in real-world settings.

We appreciate your valuable suggestion and have noted this as an important area for further exploration in the discussion section of our manuscript. Thank you again for your constructive feedback.

**2. I am confused with the definition of early death, which might vary because the majority of deaths occur from non-cancer causes. The details of non-cancer death can improve reproducibility, such as from DVT, treatment-related or drug adverse events.**

Thank you for your insightful comment regarding the definition of early death and the potential influence of non-cancer-related deaths. In our study, early death was defined as death occurring within six months after the initial pathological diagnosis of endometrial cancer, which is based on relevant literature and clinical practice. We acknowledge that the majority of early deaths may occur due to non-cancer causes, such as deep vein thrombosis (DVT), treatment-related complications, or adverse drug reactions.

This is indeed a limitation of our study, primarily due to the lack of detailed comorbidity data in the SEER database. To address this limitation, we have taken the following steps:

Emphasis on Cancer-Specific Early Mortality Risk Factors: We have adjusted the manuscript to place greater emphasis on identifying risk factors specifically associated with cancer-specific early death, rather than all-cause mortality. While our study does not include co-morbidities due to limitations in the available data, we have focused on the risk factors that are accessible and clinically relevant, which can aid in informed decision-making for patient management.

Acknowledgment of Limitations: We have explicitly acknowledged this limitation in the discussion section of our manuscript. We clarified that the study's predictive ability may be limited in real-world settings, where co-morbidities play a significant role in patient outcomes.

Future Research Directions: We propose that future research should aim to incorporate comorbidity data to enhance the study's predictive accuracy and applicability. This could involve multicenter, prospective studies or collaborations with other databases that contain more comprehensive patient health information.

**3. In inclusion criteria: The primary site of the tumor is the uterus and the first primary cancer is endometrial cancer. Were there include sarcoma?**

Thank you for pointing out the need for clarification regarding the inclusion criteria. Based on your suggestion, we have revised the manuscript to explicitly state the inclusion criteria. The updated criteria are as follows:

The inclusion site codes were C54.0 (Endometrial carcinoma of the isthmus uteri), C54.1 (Endometrial carcinoma of the corpus uteri), C54.2 (Endometrial carcinoma of the fundus uteri), C54.3 (Endometrial carcinoma of the overlapping sites of the corpus uteri), C54.8 (Endometrial carcinoma of the overlapping sites of the uterus), C54.9 (Endometrial carcinoma of the uterus, unspecified), and C55.9 (Malignant neoplasm of the uterus, unspecified). The histological codes were 8380/3 (Endometrioid adenocarcinoma), 8441/3 (Serous cystadenocarcinoma), 8480/3 (Mucinous adenocarcinoma), 8020/3 (Undifferentiated carcinoma), and 8930/3 (Adenosarcoma), according to the International Classification of Tumor Diseases, Third Edition (ICD-O-3).

Thus, endometrial stromal sarcoma (ICD-O-3 code 8930/3) was included in the study as it falls under the category of endometrial cancer with a specific histological type.

This modification ensures greater clarity and aligns with the histological types included in our study. We hope this addresses your concern. Thank you again for your valuable feedback.
